# Caveolin-1 Regulates Cellular Metabolism: A Potential Therapeutic Target in Kidney Disease

**DOI:** 10.3389/fphar.2021.768100

**Published:** 2021-12-10

**Authors:** Shilu Luo, Ming Yang, Hao Zhao, Yachun Han, Na Jiang, Jinfei Yang, Wei Chen, Chenrui Li, Yan Liu, Chanyue Zhao, Lin Sun

**Affiliations:** ^1^ Department of Nephrology, The Second Xiangya Hospital, Central South University, Changsha, China; ^2^ Hunan Key Laboratory of Kidney Disease and Blood Purification, Changsha, China

**Keywords:** caveolin-1 (Cav-1), cellular metabolism, kidney disease, oxidative stress, autophagy

## Abstract

The kidney is an energy-consuming organ, and cellular metabolism plays an indispensable role in kidney-related diseases. Caveolin-1 (Cav-1), a multifunctional membrane protein, is the main component of caveolae on the plasma membrane. Caveolae are represented by tiny invaginations that are abundant on the plasma membrane and that serve as a platform to regulate cellular endocytosis, stress responses, and signal transduction. However, caveolae have received increasing attention as a metabolic platform that mediates the endocytosis of albumin, cholesterol, and glucose, participates in cellular metabolic reprogramming and is involved in the progression of kidney disease. It is worth noting that caveolae mainly depend on Cav-1 to perform the abovementioned cellular functions. Furthermore, the mechanism by which Cav-1 regulates cellular metabolism and participates in the pathophysiology of kidney diseases has not been completely elucidated. In this review, we introduce the structure and function of Cav-1 and its functions in regulating cellular metabolism, autophagy, and oxidative stress, focusing on the relationship between Cav-1 in cellular metabolism and kidney disease; in addition, Cav-1 that serves as a potential therapeutic target for treatment of kidney disease is also described.

## 1 Introduction

Kidney disease is currently a challenging public health problem worldwide and is receiving increasing attention. The kidney is a highly metabolically active organ, and cellular energy metabolism is very important for maintaining kidney homeostasis (Gewin, 2021). In addition to inflammation, metabolic disorders are another form of diseased kidney dysfunction and play a key role in renal fibrosis (Kang et al., 2015). The scaffold protein caveolin-1 (Cav-1) on the cell membrane is a key protein to maintain energy homeostasis by regulating energy metabolism and mediating the signal transduction of glucose and lipid metabolism (Baudrand et al., 2016), which is closely associated with metabolic-related diseases such as diabetes (Bonds et al., 2019; Haddad et al., 2020), obesity (Chang et al., 2017), cardiovascular disease (Mayurasakorn et al., 2018), and cancer (Sotgia et al., 2012; Zhang Z. et al., 2020). With increased research on Cav-1, it has been demonstrated that Cav-1 plays an important role in the process of renal fibrosis (Shihata et al., 2017). However, the mechanism by which Cav-1 regulates cellular metabolism and kidney disease is not clearly understood. This review comprehensively describes the structure, expression, and regulation as well as the associated signaling pathway of Cav-1, which may be a potential drug target for metabolic-related kidney disease. Specifically, we describe the role of Cav-1 in regulating cellular glucose and lipid metabolism, cellular oxidative stress, and autophagy.

## 2 Caveolin Family

The caveolin family consists of three members, namely, caveolin-1 (Cav-1), caveolin-2 (Cav-2), and caveolin-3 (Cav-3). Cav-1 was identified in 1989 by Glenney et al. as the first family member of caveolin proteins (Glenney, 1989). They isolated a 22 kDa cytoskeleton protein from *Rous sarcoma virus*–transformed chicken fibroblasts, which was a substrate for tyrosine phosphorylation (Glenney, 1989). Subsequently, Rothberg et al. found that the 22 kDa membrane protein was part of the plasma membrane of the cell and named it caveolin (Rothberg et al., 1992). In addition, it has been demonstrated that the VIP21 protein (21 kDa) from canine renal epithelial cells (MDCK) has only eight–amino acid (aa) sequences that differ from human Cav-1. VIP21 is equivalent to caveolin in canines; thus, Cav-1 is also known as VIP21 (Glenney, 1992; Kurzchalia et al., 1992). Furthermore, caveolin was officially renamed Cav-1 in 1996 by Scherer et al. (Scherer et al., 1996). After they identified a vesicular protein–related protein, Cav-2, of about 20 kDa by microsequencing the vesicular protein–enriched membrane of adipocytes (Scherer et al., 1996). Subsequently, Tang et al. (Tang et al., 1996) found the same DNA sequence as *CAV1* in rat hearts using a gene probe, namely, *CAV3* (about 17 KDa), which was 65% identical and 85% similar to *CAV1*, whereas *CAV1* and *CAV2* had 38% identical and 58% similar DNA sequences.

*CAV1* and *CAV2* are both located on human chromosome 7q31.1, whereas *CAV3* is located on 3p25 (Engelman et al., 1998; Williams and Lisanti, 2004). Cav-1 exists in two isoforms (α and β), and each monomer has a hairpin conformation capable of dimerization (Fujimoto et al., 2000; Kogo and Fujimoto, 2000; Williams and Lisanti, 2004). Cav-1α contains 1–178 (24 kDa) residues, whereas Cav-1β contains 32–178 (21 kDa) residues (Scherer et al., 1995). In addition, the protein translation start site of Cav-1α starts from the first aa, whereas Cav-1β starts from the 32nd aa residue; thus, their N-terminal sequences are different (Scherer et al., 1995). Previous studies have suggested that these two monomers are encoded by different mRNAs (Kogo and Fujimoto, 2000). Furthermore, it is worth noting that only Cav-1α has tyrosine-14 phosphorylation sites but is not seen in Cav-1β due to the absence of a specific sequence at the N-terminus (Scherer et al., 1995; Vainonen et al., 2004). Cav-1 and Cav-2 are coexpressed in a variety of mammalian cells [such as endothelial cells (ECs), vascular smooth muscle cells, type I pneumocytes, and liver and kidney cells], whereas Cav-3 is mainly expressed not only in muscle cell but also in other cell types, such as astrocytes (Ikezu et al., 1998; Williams and Lisanti, 2004). Interestingly, it has been reported that Cav-1 is mainly in the glomeruli of the kidney (Breton et al., 1998; Sörensson et al., 2002; Moriyama et al., 2011). Furthermore, the subcellular localization of caveolins has been shown to be located in Golgi apparatus, endoplasmic reticulum (ER), mitochondria, nucleus, peroxisomes, lipid droplets (LDs) (Fridolfsson et al., 2014), and mitochondria-associated membranes (MAMs) (Sala-Vila et al., 2016). The Cav-1 protein sequence consists of four domains: an N-terminal domain (1–81 aa), an oligomerization domain (61–101 aa) including the scaffolding domain (82–101 aa), an intramembrane domain (102–134 aa), and a C-terminal domain (135–178 aa) (Filippini and D'Alessio, 2020; Root et al., 2015; Wong et al., 2020). Both the N-terminus and C-terminus of Cav-1 are exposed to the cytoplasm, and the C-terminal domain contains ubiquitination sites, whereas its N-terminal domain contains two phosphorylation sites: tyrosine-14 (Li et al., 1996b) and serine-80 (Schlegel et al., 2001). Tyrosine-14 (Y14) can be phosphorylated by the Src tyrosine kinase family (such as Src, Abl, and Fyn) (Wong et al., 2020) to regulate signaling proteins, whereas serine-80 (S80) phosphorylated Cav-1 is mainly targeted to the ER and participates in the secretion of Cav-1 (Schlegel et al., 2001). The C-terminal domain contains three palmitoylation sites (Cys133/143/156), and the palmitoylation site and the intramembrane domain (in the form of a U-shaped conformation) are embedded in the cell lipid bilayer (Hoop et al., 2012; Root et al., 2015; Park et al., 2019). In addition, the scaffold domain of Cav-1 carrying a cholesterol recognition aa consensus can bind cholesterol (Yang et al., 2014; Krishna and Sengupta, 2019) and other subtypes of caveolin, which is necessary for the formation of caveolin homo or hetero oligomers (Epand et al., 2005; Hoop et al., 2012).

Emerging evidence has shown that the scaffold domain of Cav-1 (CSD) plays a key role in tumor progression and cellular metabolic reprogramming (Bernatchez, 2020; Gopu et al., 2020). The CSD is a highly hydrophobic region composed of a 20-aa stretch of caveolin residues (Li et al., 1996a; Wong et al., 2021). It can bind proteins (such as src family kinases and G protein subunits) that contain a caveolin binding motif (CBM) consensus (Li et al., 1996a; Collins et al., 2012). The CBM includes three motifs: ΩXΩXXXXΩXXΩ, ΩXXXΩXXΩ, and the combination sequence ΩXΩXXXXΩXΩ (Ω is a Phe, Tyr, or Trp residue, X can be any aa) (Collins et al., 2012). Couet et al. found that the epidermal growth factor receptor (EGFR) kinase interacts directly with the caveolin scaffolding domain *via* its conserved caveolin binding motif, which may mediate caveolae signaling (Couet et al., 1997). However, it remains controversial whether CSD/CBM binding effectively mediates caveolae-related signaling. For instance, Collins et al. (Collins et al., 2012) stated that the CBM in most signal proteins is buried and difficult to access, and CBM sequences in caveolae-associated proteins are not abundant; thus, the interaction of CBM/CSD-mediated caveolae signaling needs to be reassessed (Collins et al., 2012). Nevertheless, Zakrzewicz et al. determined that the glycolytic enzyme enolase 1 with a CBM corecognition sequence can be transported to the cell membrane and combined with the CSD of Cav-1, which may affect cell migration and cell invasion (Zakrzewicz et al., 2014). It is worth noting that the consensus CBM of enolase 1 is not buried in the protein core but exposed on the surface of the protein, which makes it accessible for interactions with the CSD (Zakrzewicz et al., 2014). Furthermore, Okada et al. confirmed that the CSD domain of Cav-1 plays a key role in the cell cycle, migration, and proliferation of cancer cells and may provide a platform for specific signal transduction (Okada et al., 2019). In summary, caveolin may regulate other protein activities through CSD binding to the CBM, and its activity depends on binding to the CBM of other proteins.

## 3 Regulation of Caveolin-1 Expression

The expression of Cav-1 is regulated by three ways: genomic epigenetic modification, transcription, and posttranscriptional regulation mechanisms. The genomic epigenetic modification of *CAV1* can be divided into DNA methylation modification and histone modification (such as methylation, acetylation, phosphorylation, and ubiquitination) (Yan et al., 2020). Zschocke et al. (Zschocke et al., 2002) found that ectopic expression of estrogen receptor caused DNA methylation or histone deacetylation changes of *CAV1* in nerve cells, thereby regulating its mRNA expression. Subsequently, Sanders et al. (Sanders et al., 2017) found that after treatment of normal fibroblasts with transforming growth factor–β1 (TGF-β1), the mRNA expression of *CAV1* was downregulated through histone modification (H3 lysine 4 trimethylation) instead of DNA methylation, which may be associated with idiopathic pulmonary fibrosis. In addition, Yan et al. reviewed the possible mechanism of *CAV1* DNA methylation in chronic lung disease and regarded it as a possible target for early diagnosis and treatment of the chronic lung disease (Yan et al., 2020). However, whether its application as a biomarker requires further research.

The promoter sequence of *CAV1* includes three G+C-rich potential sterol regulatory elements (SREs), a CAAT sequence, a Sp1 consensus sequence (Bist et al., 1997; Dasari et al., 2006; Chen et al., 2011), and a functional peroxisome proliferator response element (Llaverias et al., 2004). Therefore, the increase in intracellular free cholesterol can regulate the mRNA expression of *CAV1* by stimulating the binding of SRE-binding protein 1 (SPEBP-1) to the cholesterol regulatory element in the *CAV1* promoter (Bist et al., 1997). Other transcription factors, including P53 (Bist et al., 2000), c-myc (Xie et al., 2003), GAT-binding factor 6 (GAT-6) (Boopathi et al., 2011), NF-κB (Thangavel et al., 2019), forkhead box O (FoxO) (van den Heuvel et al., 2005), and FoxM1 (Huang et al., 2012), can also bind to the related E-BOX of the promoter of the *CAV1* and regulate its transcription. These findings suggest that the Cav-1 may have a potential regulatory role in cellular metabolism, inflammation, and fibrosis.

Many factors contribute to the posttranscriptional regulation of *CAV1*, such as microRNA (miRNA), long noncoding RNAs (lncRNA), and circular RNA (circRNA), as well as other proteins. Among them, miRNAs, including miR-199a-5p (Zhong et al., 2020), miR-96 (Chen et al., 2020), miR-124 (Torrejón et al., 2017), miR-103/107 (Zhang et al., 2015), miR-204 (Huang et al., 2019), miR-130a (Wang et al., 2016), and miR-103-3p (Wang et al., 2021), have been demonstrated that to recognize homologous *CAV1* mRNA and cause the degradation of *CAV1* mRNA or inhibit its translation. In addition, lncRNAs, including lncRNA ANRIL (Zhong et al., 2020), lnc-BMP1-1 (Ling et al., 2020), and lncRNA IMFLNC1 (Wang J. et al., 2020), can regulate the protein expression and function of Cav-1. A recent study has shown that circRNA though some miRNAs, playing key role in posttranscriptional regulation of *CAV1* (Luo Z. et al., 2020; Zhu et al., 2020). Zhao et al. (Li et al., 2020) found that the circRNA TADA2A, contains rich binding sites of miR526b, which plays a competitive inhibitory effect, thereby releasing the inhibitory effect of miRNA526b o*n CAV1* and then increasing the expression level of Cav-1. Furthermore, some proteins, such as Cavin-1 (Hansen and Nichols, 2010; Liu et al., 2014), flotillin-1 (Vassilieva et al., 2009), and RNA-binding protein HuR (Cao et al., 2021) can also affect the protein expression and stability of Cav-1. E3 ubiquitin ligase ZNRF1 and catalase induce Cav-1 ubiquitinated (Rungtabnapa et al., 2011; Burana et al., 2016; Lee et al., 2017). In addition, the Src kinase can bind to the Tyr14 site of Cav-1 and then induces its phosphorylation, leading to instability and degradation of Cav-1 (Yoon et al., 2019). Furthermore, it has been reported that Cav-1 can be degraded by palmitic acid–induced autophagy to promote astrocyte apoptosis and inflammation (Chen et al., 2018), which indicates that the expression of Cav-1 is not only regulated by noncoding RNA but that its stability and activity are also regulated by other proteins and enzymes as well as autophagy, which, in turn, affects the function of the Cav-1 protein.

## 4 Caveolin-1 and Cellular Metabolism

### 4.1 The Role of Caveolin-1 in the Formation of Caveolae on the Cell Membrane

**FIGURE 1:**
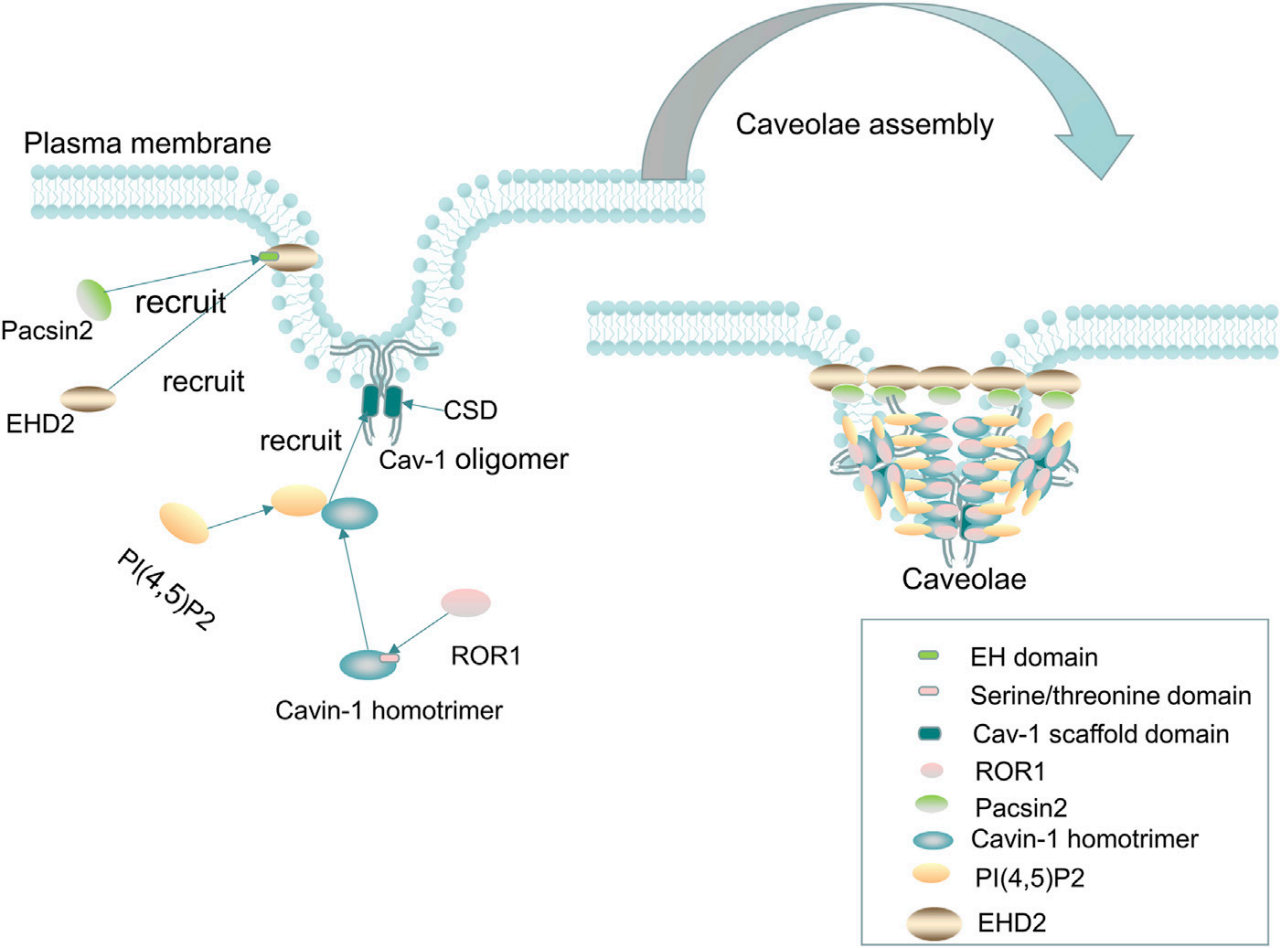
Cav-1 and caveolae. Cav-1 is essential for the formation of caveolae. The cholesterol-containing scaffold protein domain (CSD) of Cav-1 binds to cholesterol on the plasma membrane, and then, the Cavin-1 complex, EHD2, PACSIN2, and ROR1 are recruited to form mature caveolae. The recruited cavin-1 first binds to lipids [such as phosphatidylserine, and PI(4,5)P2] and then binds to the cholesterol-containing scaffold protein domain of Cav-1 to stabilize the curvature of the membrane. Two-thirds of the kinase domain of ROR1, as the scaffold protein of cavin-1 and Cav-1, binds to cavin-1, thereby promoting the binding of Cav-1 and preventing autophagy degradation of Cav-1. In addition, EHD2 is recruited to the neck of caveolae to form a loop through oligomerization, and then, Pacsin2 binds to the EH domain of EHD2 to maintain the stability of Caveolae and the distribution of Cav-1 on the plasma membrane. Without EHD2, the stability of caveolae and the distribution of Cav-1 on the plasma membrane will be difficult to maintain, resulting in dissociation of the caveolae from the plasma membrane.

An important physiological function of Cav-1 is to act as the core component of caveolae and participate in its biogenesis (Figure 1). Caveolae consist of a special membrane invagination on lipid rafts and are abundant in the plasma membrane of many mammalian cells (Parton et al., 2018). They are mainly composed of integral membrane proteins (caveolin proteins), peripheral membrane proteins (cavin proteins), and lipids (including cholesterol, sphingolipids, phosphatidylserine, glycosphingolipids, and sphingomyelin) assembled on the plasma membrane (Hirama et al., 2017), shaped like a bulb or flask, with a diameter about 50–100 nm (Cheng and Nichols, 2016). The caveolin family is the main scaffold protein involved in the formation of caveolae, which mainly refers to Cav-1; whereas in the muscle cells, it mainly refers to Cav-3 (Rothberg et al., 1992). The cavin family (including cavin-1, cavin-2, cavin-3, and cavin-4) assists in the formation of caveolae (Hill et al., 2008; Kovtun et al., 2015). Cavin-1, also known as polymerase I and transcript release factor, can bind to caveolin in a lipid-dependent manner, thereby stabilizing the curvature of caveolae (Hill et al., 2008). Cavin-2 (serum deprivation protein response) can directly bind to cavin-1 and target cavin-1 to participate in the biogenesis of caveolae by regulating the size of caveolae and inducing the expansion of caveolae-derived membrane-like tubules (Hansen et al., 2009). Cavin-3 (sdr-related gene product that binds to c-kinase) mainly regulates the membrane targeting function of caveolae (McMahon et al., 2009; Parton et al., 2018). Cavin-4, also known as muscle-restricted coiled-coil protein (MURC), is mainly restricted to expression in muscle cells and may not be important for the formation of caveolae (Parton et al., 2018). In addition, other components, such as Eps-15 homologous domain composition 2 (EHD2), PACSIN2/Syndapin II (PACSIN2), and receptor tyrosine kinase-like orphan receptor 1 (ROR1) (Yamaguchi et al., 2016) can also play an important role in modulating the function of caveolae (Parton, 2018).

The formation of caveolae mainly involves the following steps: first, caveolins, as integral membrane proteins, are synthesized in the ER, and then, Cav-1 and Cav-2 monomers are cotranslationally inserted into the membrane of the ER and form oligomerized 8S-Cav oligomers, which contain 7–14 caveolins (Han et al., 2020). The complex of oligomerized 8S-Cav oligomers is transported to the Golgi complex in the form of coat protein II (COPII) vesicle-dependent transport machinery, where Cav-1 assembles into a cholesterol-rich complex (a 70s-Cav complex composed of 18–25 8S-Cav subunits), and the 70s-Cav complex is subsequently transported and partially inserted into the plasma membrane and binds to cholesterol on the plasma membrane through its CSD domain, recruiting the cavin-1 complex, EHD2, PACSIN2, and ROR1 to form mature caveolae (Parton and del Pozo, 2013; Busija et al., 2017; Parton et al., 2018; Parton et al., 2020b).

The recruited peripheral protein cavin-1 first binds to lipids [such as phosphatidylserine and PI(4,5)P2] and then binds to the cholesterol-containing scaffold protein domain of Cav-1 to stabilize the curvature of the membrane and produce classic spherical caveolae (Hill et al., 2008). Plasma membrane insertion of EHD2 requires the binding of ATP and is oligomerized to form a ring in the neck of caveolae (Morén et al., 2012; Hoernke et al., 2017), and then, Pacsin2 binds to the EH domain of EHD2 to maintain the stability of caveolae and the distribution of Cav-1 on the plasma membrane (Hubert et al., 2020a). In addition, excessive lipid accumulation in the caveolae can cause caveolae to scission from the plasma membrane, especially in the absence of EHD2 restriction (Hubert et al., 2020b). Furthermore, EHD2 was found to regulate lipid metabolism, and loss of EHD2 caused caveolae to dissociate from the plasma membrane, while increasing fatty acid intake and promoting lipid deposition and the size of LDs (Matthaeus et al., 2020). ROR1, as a scaffold protein of cavin-1 and Cav-1, can interact with cavin-1 through its richest C-terminal serine/threonine domain, and two-thirds of the kinase domain binds to cavin-1, thus promoting the binding of Cav-1 and cavin-1 on the plasma membrane and maintaining Cav-1 expression by inhibiting lysosomal-dependent degradation and corresponding vesicle formation (Yamaguchi et al., 2016). When caveolae lack the abovementioned caveolae-related proteins to maintain their stability or subjected to mechanical stimuli (such as membrane tension or stress stimuli), Cav-1 and cavin can be released into the cytoplasm, and non-caveolae Cav-1 and cavin can recovered and reorganized into caveolae on the plasma membrane or be degraded by lysosomes (Parton et al., 2020b). In addition, non-caveola Cav-1 may also have an important physiological role independent of being a constituent of caveolae.

### 4.2 Caveolin-1 Mediates Protein Membrane Targeting, Endocytosis, and Signal Transduction

Cav-1 regulates various enzyme activities or receptor expression and targets them to the cell membrane. It has been found that the increased expression of Cav-1 can recruit glycolytic enzymes [phosphofructokinase (Vallejo and Hardin, 2005), aldolase (Raikar et al., 2006), and fatty acid translocase (CD36) (Ring et al., 2006)] and nervous system–related receptors [such as neurotrophin receptor, P2Y2 nucleotide receptor (Martinez et al., 2016), and heme oxygenase-1 (HO-1)] displaces to the cell membrane, where they directly binds the CSD of Cav-1 and then mediates downstream signal molecule transduction or fatty acid uptake (Abumrad et al., 2021), eventually affecting cellular metabolism.

Cav-1 not only affects membrane targeting of corresponding proteins but also mediates the endocytosis of some viruses, enzymes, macromolecular substances, and receptors. For example, Cav-1 participates in the endocytosis of BK virus (Moriyama et al., 2007), simian virus 40 (Damm et al., 2005), hepatitis B virus (Macovei et al., 2010), and human immunodeficiency virus (Huang et al., 2007; Mergia, 2017). However, unlike the abovementioned viruses, the SARS coronavirus entry into host cells through a novel clathrin-independent and caveolae-independent endocytic pathway (Filippini and D'Alessio, 2020; Wang et al., 2008). In addition, Cav-1 might mediate the endocytosis of receptor activin-like kinase 1 (ALK-1), which involves the bone formation protein-9 (BMP-9)/Cav-1/ALK-1 signaling pathway (Tao et al., 2020). The low expression of BMP-9 in lung ECs of mice with a knockout the *CAV1* blocks the endocytosis of ALK-1, thereby reducing the endocytosis of ALK-1–mediated low-density lipoprotein (LDL) (Tao et al., 2020). Furthermore, previous studies have found that Cav-1 can mediate the endocytosis of macromolecular protein substances, such as insulin-like growth factor (IGF)–binding protein 5 (Yamaguchi et al., 2011), albumin (Chatterjee et al., 2017), glucose transporter 4 (Glut-4) (Yuan et al., 2007), LDL, gap junction protein connexin 36 (Cx36) (Kotova et al., 2020), receptors including GM-CSF receptor β (Zsiros et al., 2019), TGF-β1 receptor (Siegert et al., 2018), and glucagon receptors (Krilov et al., 2011). Interestingly, Han et al. (Han et al., 2019) found that, in hepatocytes, Cav-1 can regulate the expression of metabolic genes induced by TGF-β. In addition, the above proteins were coexpressed with Cav-1 and could be transendocytosed, which is essential for inflammation, fibrosis, and insulin-related signal transduction, eventually affecting the cellular metabolism (Campos et al., 2019), growth, and senescence (Volonte and Galbiati, 2020). Moreover, Cav-1 also regulates T cell antigen receptor (TCR) and B cell antigen receptor (BCR) signal transduction and regulates the innate inflammatory immune response (Fiala and Minguet, 2018).

Cav-1 is also involved in the regulation of various signal transduction pathways by recruiting multiple receptors, such as receptor tyrosine kinase, G protein–coupled receptors, G proteins, protein kinases, and phosphatases (Boscher and Nabi, 2012), binds to the sequence of the CSD of Cav-1, and then positively or negatively regulates downstream signal transduction. For example, Eph receptor tyrosine kinases (EphB1 and EphA2 receptor) are positively regulated by Cav-1 and activate downstream signaling molecules, such as extracellular regulatory protein kinase (ERK) and protein kinase B (PKB or AKT) signal transduction (Vihanto et al., 2006). In addition, the insulin receptor (IR) (Chen et al., 2008) can be activated by phosphorylated Cav-1 and binds to LDL receptor–related protein 6, thereby mediating the Akt-mTORC1 signaling pathway and regulating aerobic glycolysis in cancer cells (Tahir et al., 2013).

Cav-1 negatively regulates the membrane receptors of the tyrosine kinase family [such as EGFR (Janković et al., 2017; Yang et al., 2018) and TGF-β (Lee et al., 2007; Oliveira et al., 2017)] and then modulates cell proliferation and metastasis. Similarly, G protein–coupled receptors [such as G protein–coupled receptor kinase 2 (GRK2) (Liu et al., 2017), angiotensin receptor (Ang-II) (Ishizaka et al., 1998), and P2Y2 receptor (Martínez et al., 2019)] are also regulated by Cav-1. Liu et al. (Liu et al., 2017) established a rat model of liver injury and found that the expression of phosphorylated Cav-1 increased and that Cav-1 interacted with GRK2, thereby inhibiting eNOS activity. In addition, Cav-1 promotes Ang-II–mediated Akt and EGFR signaling to cause glomerular mesangial cell hypertrophy (Umesalma et al., 2016), whereas Ang-II–mediated calcium influx is reduced (Adebiyi et al., 2014). In addition, Cav-1 can also regulates the P2Y2 receptor to increase the activity of pERK1/2 and Akt, promoting the survival of astrocytoma cells and interfering with the process of brain injury (Martínez et al., 2019). Recently, the TCR and BCR signaling pathways were found to be regulated by the abovementioned Cav-1 (Tomassian et al., 2011; Fiala and Minguet, 2018), which not only positively regulates Toll-like receptor-9 (TLR-9) to promote MyD88-mediated TRAF3 and IRF3 signal transduction (Yang et al., 2019) but also negatively regulates TLR-9 and TLR-4 to intensify the downstream inflammation cascade and promote the progression of diabetes (Zhu et al., 2017). These data indicate that Cav-1 has a huge signal transduction network, which plays an important role in cellular biological activities, including cell metabolism and growth.

### 4.3 Caveolin-1 and Lipid Metabolism

#### 4.3.1 Caveolin-1 and Biogenesis of Lipid Droplets

LDs are organelles with special structures that are ubiquitous in most eukaryotic cells and are involved in regulating energy metabolism and maintaining cellular homeostasis (Walther and Farese, 2012; Olzmann and Carvalho, 2019; Henne et al., 2020). The hydrophobic core of LDs is composed of triacylglycerols (TGs) and sterol esters (SEs) as neutral lipids and is surrounded by an ER-derived phospholipid monolayer, which is decorated with integral and peripheral proteins (Bersuker and Olzmann, 2017; Gao et al., 2019). The synthesis of neutral lipids is the first step in the formation of LDs. In mammalian cells, it is mainly catalyzed by ER diacylglycerol acyltransferase (DGAT) enzyme (DGAT1/2) and cholesterol acyltransferase (ACAT) enzyme (ACAT1/2) synthesis; among them, DGAT catalyzes the synthesis of TGs, whereas ACAT catalyzes the synthesis of SEs (Walther and Farese, 2012; Walther et al., 2017). The newly synthesized neutral lipid forms a lens-like structure between the leaflets of the ER bilayer and then can gradually fuse into larger and more stable lenses and buds from the ER to form nascent LDs (Renne et al., 2020). During this process, a continuous supply of phospholipids from the ER is needed to assist in the further expansion of LDs (Renne et al., 2020). In addition, some specific proteins are transported to the surface of LDs to promote their growth and expansion, such as glycerol-3-phosphate acyltransferase 4, DGAT2, adipose triglyceride lipase (ATGL), and other proteins, reaching the surface of LDs through ER-LD membrane bridges (Wilfling et al., 2014; Walther et al., 2017). Cytoplasmic proteins such as Perilipin family proteins and CCT1 are targeted to LDs through their hydrophobic domains (Walther et al., 2017). The perilipin family proteins PLINs1-5 are the main LD-related proteins and mainly refer to PLIN1 (perilipin A), PLIN2 (adipophilin), PLIN3 (TIP47), PLIN4 (S3-12), and PLIN5 (MLDP) (Itabe et al., 2017), which may regulate intracellular lipolysis. PLIN1 is highly expressed in mature adipocytes and forms a complex with α/β-hydrolase D5 (ABHD5) (CGI-58) on the surface of LDs under basic conditions (Sztalryd and Brasaemle, 2017). When PLIN1 is phosphorylated by cAMP, phosphorylated CGI-58 dissociates from it and binds to phosphorylated ATGL to activate triglyceride hydrolysis (Sztalryd and Brasaemle, 2017). In addition, cytoplasmic hormone-sensitive lipase (HSL) is phosphorylated by PKA and can be transported to the surface of LDs to participate in lipolysis (Frühbeck et al., 2014; Itabe et al., 2017). Furthermore, herein, we describe an important integral membrane protein, Cav-1, related to LD biogenesis and metabolism.

**FIGURE 2:**
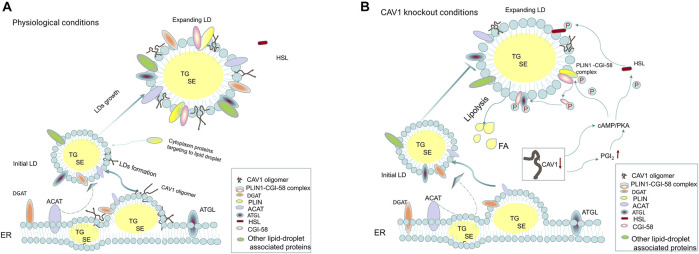
Cav-1 and lipid droplet biogenesis. **(A)** Under physiological conditions, the initial step of lipid droplets (LDs) formation is the synthesis of neutral lipids (TGs and SEs) by DGAT and ACAT on the ER, which serve as the core of LDs. As the LDs gradually expand, LD-associated proteins and ER-derived monolayer phospholipids are distributed around neutral lipids, promoting the initial lipid droplets to become enlarged lipid droplets. Cytoplasmic proteins, such as PLIN family proteins, are involved in regulating lipid droplet metabolism and can target the lipid droplet surface, among which PLIN1 binds to CGI-58 protein to form the PLIN1-CGI-58 complex. The newly synthesized caveolin-1 is inserted into the bilayer of the ER and fused and transferred to the LDs. Caveolin-1 is involved in LD biosynthesis; on the one hand, it may affect the distribution of phospholipids on the surface of LDs, and on the other hand, it is essential for the expansion of LDs. **(B)** When *CAV1* is knocked out in cells (such as adipocytes and endothelial cells), the following processes may be affected: 1. growth and expansion of new lipid droplets are hindered; 2. the reduction in caveolin-1 expression can increase the phosphorylation of HSL and PLIN1 in a cAMP-dependent manner to enhance lipolysis. The latter is regulated by the PGI2/cAMP/PKA pathway, leading to phosphorylation of PLIN1 and CGI-58. Phosphorylated CGI-58 dissociates from PLIN1 and activates ATGL to co-activate lipolysis.

Cav-1 is one of the resident proteins of LDs and is involved in LD biogenesis (Pezeshkian et al., 2018). Roy et al. proposed that caveolin plays a cholesterol-trafficking role, and dominant-negative caveolin (CavDGV) can inhibit the signal transduced by H-Ras and change the distribution of cholesterol (Roy et al., 1999). Pol et al. showed that mutant caveolin protein (Cav-3DGV) accumulates in the ER and is targeted to the limiting membrane of LDs (Pol et al., 2001). However, Blouin et al. found that the expression level of Cav-1 affects the size of LDs, and in adipocytes lacking *CAV1*, the species of phospholipids on the LD surface are reduced, and only small LDs can be formed (Blouin et al., 2010). The accumulation of Cav-1 in the ER is targeted to LDs, which is related to its hydrophobic domain, especially its COOH-terminal domain sequence (Ostermeyer et al., 2004). Robenek et al. proposed a LD biogenesis model and hypothesized that the process of LD formation involves the synthesize lipid on the ER membrane, accumulation in the center of the bilayer to form a disc, and separation of Cav-1 from the ER membrane into LDs (Robenek et al., 2004). Furthermore, they stated that Cav-1 is not limited to the outer membrane monolayer around the LD but also present in the core of LDs (Robenek et al., 2004). Conversely, Cav-1 tends to be distributed in the lipid bilayer at the edge of the triolein lens and does not affect the curvature of the lipid lens but affects the distribution of surface neutral lipids and phospholipids on the LD surface (Pezeshkian et al., 2018). Moreover, *CAV1* null adipocytes/fat pads increase protein kinase A (PKA) activity, which leads to phosphorylation of HSL and perilipin followed by the activation of lipolysis (Cohen et al., 2004). In addition, *CAV1* gene knockout in the ECs increase the autocrine activity of prostaglandin I2 (PGI2), which acts as a stimulus to activate the cAMP/PKA pathway to promote the phosphorylation of HSL to increase lipolysis and reduce the formation of LDs, but it does not reduced triglyceride synthesis or fatty acid uptake (Kuo et al., 2018), suggesting that Cav-1 may play an important role in LD accumulation (Figure 2). Interestingly, previous studies have shown that exogenous addition of fatty acids may act as a signal to promote caveolin targeting LDs in nonadipocyte types (Liu et al., 2004; Pol et al., 2004). Similarly, Le Lay et al. found that, in adipocytes, an increase in exogenous cholesterol promotes the activation of Src, which triggers dynamin-dependent caveolae budding and trafficking of Cav-1 from the plasma membrane to LDs (Le Lay et al., 2006). Recently, the role of LD organelles (such as the ER, mitochondria, peroxisomes, lysosomes, and nucleus) in regulating lipid metabolism has attracted more attention (Barbosa et al., 2015; Barbosa and Siniossoglou, 2017; Suzuki, 2017; Geltinger et al., 2020). Yokomori et al. proposed that the transport of Cav-1 from the ER to LDs may be related to liver cirrhosis, and the connection between the ER and LDs may be a potential mechanism (Yokomori et al., 2019). Because Cav-1 is an important component of the membrane of each suborganelle, the role of Cav-1 in the communication of organelles and lipid metabolism may need to be addressed in the future.

#### 4.3.2 Caveolin-1 Regulates Cholesterol Homeostasis

Cav-1 has a high affinity for cholesterol and participates in the regulation of cellular cholesterol homeostasis, including cholesterol transport, cholesterol signal transduction, and cholesterol metabolism (Fielding and Fielding, 1995; Smart et al., 1996; Ikonen and Parton, 2000). Cholesterol metabolism includes endogenous synthesis, exogenous uptake, esterification, and efflux (Chang et al., 2006). The endogenous synthesis of cholesterol is regulated by SREBPs, which have three subtypes, namely, SREBP1a, SREBP1c, and SREBP2 (DeBose-Boyd and Ye, 2018). Among them, SREBP2 is mainly involved in cholesterol metabolism, SREBP1c participates in fatty acid synthesis, and SREBP1a regulates both cholesterol and fatty acid synthesis (Luo J. et al., 2020). SREBP2 is considered to be a key mediator of cholesterol biosynthesis, is synthesized in the ER, and then interacts with insulin-induced gene-1 protein (INSG-1) and SREBP cleavage activation protein (SCAP). When cholesterol in the ER is exhausted, INSG-1 can be degraded by lysosomes, and the SREBP2 and SCAP complexes are sorted into COPII-coated vesicles and escorted to the Golgi apparatus, where SREBP2 is processed by site 1 protease (S1P) and site 2 protease (S2P) cleavage, exposing the active N-terminal domain, and enters the nucleus to combine with the promoter regulatory element (SRE), thereby activating the transcription of cholesterol synthesis genes (Afonso et al., 2018; Brown et al., 2018; Yan et al., 2021). Interestingly, a previous study has shown that Cav-1 plays an important role in the transport of intracellular cholesterol, transporting newly synthesized cholesterol from the ER to caveolae and participating in the composition of the cell membrane (Smart et al., 1996). In skin fibroblasts, the expression of Cav-1 is also affected by cellular cholesterol levels, SREBP1 can inhibit the transcription of *CAV1*, and low levels of Cav-1 can further affect cholesterol homeostasis (Bist et al., 1997). As described in the review by Bosch et al., the loss of *CAV1* in cells (such as fibroblasts) leads to the accumulation of cholesterol on the ER, which may be transported to mitochondria *via* MAMs, and the accumulation of cholesterol on mitochondria leads to mitochondrial dysfunction and increased reactive oxygen species (ROS) production, eventually affecting cellular metabolism (Bosch et al., 2011). Pol et al. found that, in hamster kidney (BHK) cells transfected with mutant *CAV3* (Cav-3DGV), a dominant negative mutant, intracellular cholesterol transport to the cell membrane was blocked, resulting in the redistribution of cholesterol, which was characterized by reduced plasma membrane cholesterol, accumulation of free cholesterol in the lysosomes, and reduced cholesterol efflux and synthesis (Pol et al., 2001). Furthermore, Frank et al. demonstrated that the loss of *CAV1* in mouse embryonic fibroblasts (MEFs) and mouse peritoneal macrophages leads to an increased accumulation of cholesterol in the ER, and the accumulation of cholesterol can increase the activity of acyl-CoA-cholesterol acyltransferase (ACAT) and reduce the synthesis of free cholesterol (Frank et al., 2006). ACAT (including ACAT1/2) is an enzyme that is involved in the esterification of cholesterol, and its role is to esterify free cholesterol and prevent free cholesterol from accumulating freely in cells and then causing lipotoxicity (Luo J. et al., 2020). Recently, Xu et al. reported that overexpression of ACAT1/2 in 3T3-L1 adipocytes promoted the colocalization of Cav-1 and free cholesterol on the surface of LDs, impairing the function of adipocytes and cholesterol homeostasis (Xu et al., 2019). This information indicated that Cav-1 is closely related to cholesterol homeostasis and that Cav-1 may play a key intermediate mediator role in cellular cholesterol metabolism.

To maintain cholesterol homeostasis, cells not only esterify free cholesterol but also need to remove excess cholesterol through efflux. Current knowledge indicates that the scavenger receptor-B1 (SR-BI) and ATP-binding cassette (ABC) transporter family proteins ABCA1 and ABCG1 can escort cholesterol in macrophages to various extracellular receptors (such as apoA-I, HDL, and LDL) and then transport them to the peripheral blood, thereby reducing the burden of cholesterol in the cell (Sharma and Agnihotri, 2019; Plummer et al., 2021). Furthermore, it has been found that Cav-1 and SR-BI are simultaneously upregulated in differentiated THP-1 macrophages, and their coexpression promotes the efflux of cholesteryl esters to HDL (Matveev et al., 1999). However, another study found that SR-BI–mediated selective cholesteryl ester uptake in human embryonic kidney 293 or Fischer rat thyroid (FRT) cells was not affected by Cav-1 expression (Wang et al., 2003). In addition, deficiency *CAV1* gene in macrophages had a slight effect on ABCA1-mediated cellular cholesterol efflux to apoA-I but did not affect SR-BI and ABCG1-mediated cholesterol efflux to the HDL receptor (Frank et al., 2006), which indicates that the Cav-1 can regulate intracellular cholesterol efflux in different cell lines.

### 4.4 Caveolin-1 and Glucose Metabolism

It is currently known that Cav-1 is involved in regulating glucose metabolism (Nwosu et al., 2016; Gopu et al., 2020). The loss of *CAV1* causes impairs glucose homeostasis and dyslipidemia, which is reversed by downstream aldosterone (MR) inhibition (Baudrand et al., 2016). Significantly increased *CAV1* mRNA in the peripheral blood of patients with metabolic syndrome has been observed (de Souza et al., 2020). In contrast, Luo et al. found that mRNA expression of *CAV1* is decreased in patients with type 2 diabetes, which may occur through direct binding of miR-103 to *CAV1* (Luo M. et al., 2020). Furthermore, Fachim et al. demonstrated that changes in lifestyle (such as exercise or diet) can cause DNA methylation in the *CAV1* transcript region and affect its expression in adipose tissue and peripheral blood cells in patients with type 2 diabetes (Fachim et al., 2020). They observed that decreased expression of Cav-1 in adipose tissue but increased expression in peripheral blood (Fachim et al., 2020). However, whether the changes of Cav-1 in other organs of patients with type 2 diabetes will affect other signaling proteins to regulate the progression of diabetes is worthy of further investigation. Furthermore, current evidence from various studies has shown that Cav-1 affects glucose metabolism by regulating a variety of glucose uptake transporters (Yuan et al., 2007; Elvira et al., 2013; Varela-Guruceaga et al., 2018). Lee et al. found that the expression of Cav-1 affects SGLT1 receptor–mediated glucose uptake on renal tubular epithelial cells by affecting the cAMP/Epac/PKA signaling pathway (Lee et al., 2012). In addition, the enhanced expression of ERK, p38MAPK, and NF-κB can increase the activity of SGLT1 and promote its binding to Cav-1 in renal tubular epithelial cells to increase glucose uptake (Lee et al., 2012). In addition, Cav-1 can upregulate the expression of SGLT1 (Elvira et al., 2013), indicating that Cav-1 may play a key role in glucose uptake by renal tubules, but the role of blood glucose in diabetes may need to be studied in the future.

Moreover, Cav-1 can regulate GLUT4 to mediate glucose uptake, which is related to insulin-related signaling pathways (Yuan et al., 2007). Long-term high glucose stimulates adipocytes and reduces the sensitivity of Cav-1 to insulin and inhibits IR and PKB (AKT-2) phosphorylation, which affect the GLUT4 in the intracellular glucose storage vesicles to transport to the plasma membrane caveolae and bind to Cav-1 and subsequently mediate glucose uptake (Palacios-Ortega et al., 2016). In contrast, in a high-glucose environment, the caveolae-related coiled-coil protein (NECC2) is highly expressed on adipocytes, which is closely linked to insulin-related glucose uptake by triggering insulin induction of NECC2 transport to the cell surface and binding to Cav-1 (Trávez et al., 2018). At this point, the IR binds to the scaffold domain of Cav-1 and then activates the PI3K/Akt signaling pathway (Trávez et al., 2018). However, Cav-1 regulates glucose metabolism in tumor cells mainly by regulating glucose transporter 3 (GLUT3/SLC2A3) uptake of glucose, which leads to increased aerobic glycolysis and increases intracellular ATP production, thus maintaining the growth and survival of tumor cells (Ali et al., 2019). Further investigations have shown that the abnormally increased expression of Cav-1 in tumor cells is related to the methylation of CpG sites, and the upregulation of Cav-1 expression then increases the activity of HMGA1 and promotes the translocation of HMGA1 into the nucleus, where HMGA1 binds to the promoter of GLUT3 and promotes its transcription (Ha et al., 2012). In addition, Cav-1 also affects mitochondrial function, increasing ATP production and inhibiting phosphorylation of AMPK at the Thr172 residue and modulating autophagy by the AMPK-TP53/p53 pathway in cancer cells (Ha and Chi, 2012). Further research demonstrated that the hypoxia response elements (HREs) of hypoxia inducible factor-1α (HIF-1α) bind to the promoter of *CAV1* under hypoxic conditions and inhibit the transcript level of *CAV1*, reducing the translocation of GLUT4 to the plasma membrane and resulting in decreased glucose uptake (Varela-Guruceaga et al., 2018). In addition, glucose uptake mediated by GLUT4 membrane translocation is related to AMP-activated protein kinase (AMPK), which is a key sensor of glucose metabolism (Hu et al., 2017). Therefore, it is worth noting that Cav-1 plays an indispensable role in regulating cellular metabolism and may be a novel or important target in metabolic-related diseases.

### 4.5 Caveolin-1 and Autophagy

Autophagy plays an important role in cellular metabolism, and a recent study reported that autophagy are closely related to cellular metabolism and cell survival (Tan and Miyamoto, 2015). Participation in the regulation of autophagy is one of the functions of Cav-1. Cav-1 can bind to the autophagy-related protein 5 (ATG-5), ATG12, and ATG5-ATG12 complex in lung epithelial cells and inhibit the formation and function of autophagosomes (Chen et al., 2014). When the cells are under stress (such as starvation), knockout of *CAV1* gene in MEFs can promote the formation of late autophagy lysosomes, enhance autophagic flux and promote cell survival, and it is involved in tumor progression (Shi et al., 2015). Deficiency of *CAV1* gene in human aortic ECs causes connexin-43 and ATG5 from the plasma membrane to combine with several ATGs (such as ATG12, ATG16, and IP3R), which are involved in the initial steps of autophagosome formation to promote the formation of autophagosomes, thus inhibiting vascular inflammation and arterial wall macrophage infiltration (Zhang X. et al., 2020). However, in cisplatin-treated lung cancer cells, silencing *CAV1* with siRNA inhibits the activation of Rho-related coiled-coil kinase 1 (ROCK1), thereby affecting Parkin-related mitochondrial autophagy and protecting against mitochondrial apoptosis and functional damage (Liu et al., 2020). In addition, under oxidative stress, Cav-1 phosphorylates at tyrosine-14, binds to beclin-1, and promotes its translocation to mitochondria, promoting mitochondrial autophagy (Nah et al., 2017). Recent studies have shown that the HIF-1α–Cav-1 signaling axis mediates autophagy to regulate cellular metabolism and promote the survival and metastasis of breast cancer cells (Wang N. et al., 2020). In addition, the close relationship between Cav-1 and autophagy plays an important role in regulating lipid metabolism. Bai et al. found that, in human umbilical vein ECs treated with high glucose, autophagy degradation of Cav-1 mediated by the AMPK/mTOR/PIK3C3 pathway is inhibited, thereby increasing the expression of Cav-1 and cavin, and then, LC3 is recruited and bound, subsequently inhibiting Cav-1-CAVIN1-LC3B-mediated autophagy degradation of Cav-1. This process eventually manifests as the increased expression of Cav-1 to promote the formation of caveolae and mediates the increase in LDL endocytosis (Bai et al., 2020). Interestingly, Xue et al. found that overexpression of *CAV1* in L02 cells treated with alcohol and oleic acid can inhibit the Akt/mTOR pathway, thereby activating autophagy and alleviating cellular lipid deposition (Xue et al., 2020). The above information indicates that Cav-1 has different roles in the regulation of autophagy and cellular metabolism in different cell lines.

### 4.6 Caveolin-1 and Oxidative Stress

Oxidative stress is the key factor in cellular metabolism (Liu et al., 2019). It has been demonstrated that Cav-1 regulates oxidative stress and participates in the process of cellular life. Under oxidative stress, the expression of Cav-1 is increased in nucleus pulposus cells and related to premature senescence, whereas silencing the expression of *CAV1* can reduce the protein expression of p53 and p21 to protect cells from senescence (Ding et al., 2017). In contrast, under oxidative stress, Cav-1 is abundantly expressed in chondrocytes and transferred from the plasma membrane to the nucleus to participate in the repair of DNA damage (Goutas et al., 2020). In addition, overexpression of *CAV1* in rhabdomyosarcoma cells, the cell cycle is blocked in the G2/M phase, which is accompanied by the reduced expression of p21, p16, and cleaved caspase-3, whereas the production of catalase is increased; therefore, Cav-1 enhances DNA repair and protects against cellular senescence and apoptosis from oxidative stress (Codenotti et al., 2021). However, interestingly, Goutas et al. did not observe a displacement of Cav-1 and the DNA damage repair in patients with osteoarthritis (Goutas et al., 2020). Thus, the role of Cav-1 in DNA repair in cells requires further confirmation.

There is a closed relationship between oxidative stress and inflammation. Studies have shown that the Cav-1 regulates oxidative stress and affects inflammation. Wang et al. found that high-fat diet-fed ApoE^−/−^ mice with atherosclerosis show increased activity of JNK-related signals, oxidative stress, and inflammation, whereas these changes were rescued in mice with a double knockout of ApoE and *CAV1* (Wang et al., 2018). In contrast, in mice with liver injury induced by carbon tetrachloride (CCl_4_), *CAV1* gene knockout can aggravate oxidative stress and activate the TGF-β signaling pathway and the production of proinflammatory factors, such as IL-1β and IL-6, leading to liver fibrosis (Ji et al., 2018). Furthermore, in E11 murine kidney podocytes, Cav-1 promotes the production of antioxidant enzymes and inhibits the oxidative stress response induced by H_2_O_2_, alleviating the inflammatory damage of podocytes (Chen et al., 2017). Conversely, Cav-1 also affects the energy conversion of cells (Fernández-Rojo et al., 2012). Recently, Shao et al. found that knockdown of *CAV1* with shRNA in pancreatic stellate cells promotes the production of ROS, and the production of ROS further reduces the expression of Cav-1 (Shao et al., 2020). This Cav-1-ROS positive feedback induces the conversion of cell energy metabolism to glycolysis, and the products of glycolysis promote cell energy production through mitochondrial oxidative phosphorylation, which further promotes the proliferation of pancreatic cancer cells (Shao et al., 2020). This information indicates that Cav-1 plays an important role in regulating oxidative stress and inflammation by affecting cellular metabolism.

### 4.7 Other Functions of Caveolin-1: Mechanical Sensing and Vesicle Transport

Cav-1, as the main component protein of caveolae, is involved not only in regulating metabolism but also in mechanical transduction and vesicle transport. Cav-1 phosphorylation is essential for caveolae to perform signal transduction, endocytosis (Nabi and Le, 2003) and lipid transport (Pilch and Liu, 2011); enzymes (Coelho-Santos et al., 2016), viruses (Xing et al., 2020), and LDL (Gerbod-Giannone et al., 2019) enter the cell through caveolae-dependent endocytosis, which is related to phosphorylation of Cav-1. Caveolae act as a cell membrane sensor (Parton and del Pozo, 2013) and mechanical sensor (Sinha et al., 2011), which can quickly adapt to sudden and acute mechanical stress stimulation (Sinha et al., 2011). When the cell responds to a variety of stimuli, such as osmotic/stretch, shear, ultraviolet, and oxidative stimuli, the tension of the cell membrane increases, which promotes caveolae disassembly (Parton et al., 2020a). The above stimuli cause caveolae flattening and may eventually induce cavin dissociation, changes in lipid distribution on the cell membrane, and Cav-1 tyrosine phosphorylation (Parton et al., 2020a). Phosphorylated Cav-1 can regulate actin to detach caveolae from the plasma membrane and enter the cytoplasm (Zimnicka et al., 2016) and then traffic to LDs, which may be involved in cell lipid metabolism (Matthaeus and Taraska, 2020). It is particularly noteworthy that the relationship between Cav-1 and lipid metabolism has aroused widespread research interest. Recent studies have found that Cav-1 regulates lipid metabolism and participates in kidney-related diseases (Chen et al., 2016; Mitrofanova et al., 2019). Therefore, we focused on the relationship between Cav-1 regulation of cellular metabolism and the kidney.

## 5 Caveolin-1 and Kidney Disease

### 5.1 Acute Kidney Disease

Cav-1 is involved in the pathophysiology of acute kidney injury (AKI). Zager et al. found that the expression of Cav-1 was increased in ischemia ± reperfusion–induced AKI mice and verified that the destruction of the caveolae in the damage of renal tubular epithelial cells, which causes cholesterol and Cav-1 are separated from the plasma membrane, leading to free cholesterol deposits in the lumen of the renal tubules and an increased level of urine Cav-1 (Zager et al., 2002). Accordingly, Cav-1 was considered a possible biomarker of AKI (Zager et al., 2002). In addition, proximal renal tubular injury leads to increased destruction of caveolae, and Cav-1 translocates into the cytoplasm; activates the expression of PDGFR-β, EGFR, and Rho guanosine triphosphatase (GTPase) signaling proteins; and participates in the process of renal tubular cell regeneration (Mahmoudi et al., 2003; Fujigaki et al., 2007). Likely, in ischemic reperfusion AKI mice treated with EPO, a significant increase in the expression of Cav-1 in blood and kidney tissue is observed (Kongkham et al., 2016). In addition, Cav-1 has also been found to be highly expressed in apoptotic tubular cells, although it remains controversial whether Cav-1 plays a role in promoting repair or apoptosis in AKI (Mahmoudi et al., 2003). Notably, Moore et al. reported that Cav-1 is a tissue fibrosis inhibitor, and its genetic polymorphisms are associated with renal transplantation fibrosis and allogeneic transplantation failure (Moore et al., 2010). Furthermore, another recent study has demonstrated increased expression of Cav-1 in the serum of patients with kidney transplant, which associated with a decreased incidence of tubulointerstitial rejection (Emmerich et al., 2021). From the above information, it can be seen that Cav-1 has different roles in AKI, and its detailed mechanism still lacks experimental verification.

### 5.2 Glomerulus Nephritis

Although glomerulonephritis is an immune-mediated disease, recent studies have shown that consistent changes in the kidney transcriptome are consistent with the metabolic reprogramming of different forms of glomerulonephritis (Grayson et al., 2018), which may indicate that abnormal cellular metabolism also plays an important role in this disease. Tamai et al. showed, for the first time, that caveolae are present in mesangial cells and the Cav-1 is located on caveolae, as detection by electron microscope (Tamai et al., 2001). Furthermore, they demonstrated that Cav-1 can bind to PDGF receptors to mediate the PDGF pathway and modulate mesangial proliferative glomerulonephritis (Tamai et al., 2001). In addition, Ostalska-Nowicka et al. verified that the expression of Cav-1 in parietal epithelial cells is significantly lower in children diagnosed with focal segmental glomerulosclerosis and lupus glomerulonephritis than in those with minimal change disease, Schönlein-Henoch glomerulopathy, or in controls (Ostalska-Nowicka et al., 2007). Furthermore, the high expression level of Cav-1 in glomerular ECs is positively correlated with proteinuria, and it is suggested that Cav-1 mediates EC endocytosis of albumin and participates in the progression of glomerular-related diseases (Moriyama et al., 2011; Moriyama et al., 2017). Although current studies suggest that Cav-1 may regulate the progression of glomerular associated diseases by endocytosing of macromolecules (cholesterol or albumin), the mechanism by which Cav-1 modulates the development of glomerular diseases requires further study.

### 5.3 Diabetic Kidney Disease

Previous studies have shown that Cav-1 has antifibrotic properties by regulating cell proliferation, migration and adhesion, as well as inhibiting the TGF-β signaling pathway in diabetic kidney disease (DKD) (Gvaramia et al., 2013; Shihata et al., 2017; Van Krieken and Krepinsky, 2017). In addition, Cav-1 might also regulate glucose uptake and mediate the endocytosis of urinary albumin; therefore, it is considered a potential therapeutic target for DKD (Van Krieken and Krepinsky, 2017). Arya et al. found that, in rats with diabetic nephropathy, the highly expressed Cav-1 can bind to nitric oxide synthase (eNOS) and inhibit its signal transduction, thereby reducing the production of NO and eventually leading to increased level of serum urea nitrogen, blood creatinine and urine protein, whereas the above changes can be reversed by Cav-1 inhibitors (Arya et al., 2011). Conversely, in renal mesangial cells treated with high glucose, Cav-1 phosphorylated by Src kinase can activate RhoA, which may be related to the development of glomerular matrix accumulation in DKD (Wu et al., 2014). Xie et al. showed that, in mesangial cells treated with high glucose, RhoA/Rock promotes the translocation of NF-kB into the nucleus to increase the transcription level of inflammatory factors such as ICAM-1, TGF-β1, and FN and ultimately leads to the production of mesangial cell matrix (Xie et al., 2013). Furthermore, high glucose has been shown to induce an increase in the production of mitochondrial ROS (mtROS) and vascular endothelial growth factor (VEGF), and mtROS in glomerular ECs, which activates Src kinase to phosphorylate Cav-1, leading to increased albumin endocytosis and massive proteinuria (Xie et al., 2013). Furthermore, under high-glucose conditions, phosphorylation of Cav-1 upregulates the expression of TLR-4 and promotes the secretion of pro-inflammatory factors, such as TNF-α, IL-6, IL-1β, and MCP-1 in podocytes and accelerates the process of DKD (Sun et al., 2014b). More importantly, diabetic mice with deficiency of *CAV1* gene in mesangial cells showed a significant inhibitory effect on the PKCβ1/ROS/RhoA/Rho-kinase signaling pathway; the expression of TGFβ1, FN, and collagen I is reduced, whereas the expression of AMPK is upregulated (Zhang et al., 2012; Guan et al., 2013). However, how Cav-1 regulates AMPK expression affects the development of DKD is unknown. Another study found that *CAV1* knockout in mesangial cells of diabetic mice upregulates the protein expression of follistatin, which neutralizes and inhibits activin, ultimately decreasing proteinuria, glomerular sclerosis, and extracellular matrix accumulation (Zhang et al., 2019). SMPDL3b is an enzyme related to lipid metabolism, which can downregulate the expression of ceramide-1-phosphate and affect the phosphorylation of AKT, leading to podocyte damage (Mitrofanova et al., 2019). In mice carrying a specific knockout of the SMPDL3b in podocytes, the Cav-1 combines with IRB to transduce insulin signals and phosphorylate Akt, which reduces podocyte damage and delays the progression of DKD (Mitrofanova et al., 2019). These data suggest that Cav-1, a cell metabolism related molecule, plays a critical role in kidney injury in DKD.

### 5.4 Clear Cell Renal Cell Carcinoma

Metabolic reprogramming in clear cell renal cell carcinoma (RCC) has been recognized (Wettersten et al., 2017). It has been reported that Cav-1 inhibits breast cancer stem cells through metabolic reprogramming (Wang S. et al., 2020). Recent evidence has shown that Cav-1 is considered one of the possible prognostic biomarkers of clear cell RCC (Waalkes et al., 2011). Cav-1, which is increased significantly in RCC kidney tissue, can regulate the growth and metastasis of cancer cells (Joo et al., 2004; Steffens et al., 2011). In addition, the expression of Cav-1 and phosphorylated ERK-1/2 in local RCC tissues is also considered to be a predictor of metastasis of RCC (Campbell et al., 2013). The Cav-1/AKT/mTOR axis has been shown to promote the proliferation of cancer cells and vascular metastasis (Campbell et al., 2008). Recently, Zhang et al. found that Cav-1 binds to oxidized low-density apolipoprotein receptor 1 (LOX-1) to induce lipid deposition and promote tumor cell proliferation, but this effect can be reversed by celastrol (Zhang et al., 2021). Although Cav-1 participates in the metabolism of cancer cells and regulates the survival of cancer cells, the relationship between Cav-1 and RCC merits in-depth study.

## 6 Therapeutic Promising

Because Cav-1 plays an important role in cellular metabolism and activities related to cellular life, especially the development of various kidney diseases, recent studies targeting Cav-1 for the treatment of various diseases, especially kidney diseases, have become a research hotspot (Table 1). Here, we focus on some chemical compounds, drugs, and various extracts from traditional Chinese medicine, which target Cav-1 or its associated signaling pathway and summarize their application in kidney diseases.

**TABLE 1:** Potential therapeutic target of Cav-1 in kidney disease.

Category	Compound/Effector	Mechanism	Major findings in kidney disease	References
Chemical compounds	Methyl-beta-cyclodextrin	Destroy caveolae	1. Mesangial cells: the production of collagen I and fibronectin is reduced, which reduces mesangial expansion and mesangial cell hypertrophy.	Moriyama et al. (2017), Peng et al. (2007), Zhang et al. (2007)
			2. Endothelial cells: protect the filtration function of the kidneys and reduce proteinuria.	
	Filipin	Destroy caveolae	Mesangial cells: mainly reduce the internal pressure of the glomerulus and relieve glomerular sclerosis.	Peng et al. (2007), Zhang et al. (2007)
	Daidzein	Inhibit the expression of Cav-1	1. Inhibit the Cav-1-eNOS pathway, increase kidney NO production, and reduce blood urea nitrogen, serum creatinine, urine protein, and collagen content in diabetic rats	Arya et al. (2011), Meng et al. (2017), Tomar et al. (2020)
			2. Renal tubular cells: anti-inflammatory, antioxidant, reduction of urine protein, creatinine, and urea nitrogen.	
Extract from Traditional Chinese Medicines	Curcumin	Inhibition of Cav-1 Y14 phosphorylation	Podocytes: reduce the damage caused by pro-inflammatory factors to podocytes and alleviate oxidative stress and apoptosis of podocytes.	Sun et al. (2014a)
	Salidroside	Inhibit Cav-1 Y14 phosphorylation	Endothelial cells: protect the filtration function of the kidneys and reduce proteinuria.	Wu et al. (2016)
	Catalpol	Inhibit Cav-1 Y14 phosphorylation	Reduce kidney damage and inhibiting mesangial cell proliferation by improving lipid metabolism, IGF-1 signaling.	Bai et al. (2019), Zhou et al. (2014)
Rock inhibitor	Fasudil	Inhibit Cav-1/RhoA and VEGF/Cav-1 pathways	1. Mesangial cells: reduce the production of ICAM-1, TGF-β1, and FN and alleviate renal fibrosis.	Huang et al. (2021), Jin et al. (2015), Xie et al. (2013)
			2.Podocytes: reduce the inflammatory damage of IL-6 and MCP-1 to podocytes.	
Noncoding RNA	miR-204	Inhibit Cav-1/TRPM3-mediated autophagy	miR-204 indirectly inhibits TRPM3-mediated downstream LC3B-related autophagy through Cav-1, thereby inhibiting the progression of clear cell renal cell carcinoma.	Hall et al. (2014)
	CircAKT1	Sponge miR-338-3p and upregulate Cav-1 expression	Promote the proliferation, migration, invasion, and epithelial-mesenchymal transition (EMT) of clear cell renal cell carcinoma cells.	Zhu et al. (2020)

### 6.1 Caveolin-1 Inhibitor

#### 6.1.1 Chemical Compound

Methyl-β-cyclodextrin (MβCD), filipin, and daidzein are the more common inhibitors of Cav-1 (Woodman et al., 2004; Peng et al., 2007; Gao et al., 2014). Recent studies have shown that the effects of these drugs on the functions of Cav-1 may be secondary to the disruption of caveolae. MβCD and filipin can destroy the structure of caveolae and reduce Cav-1 phosphorylation and downstream signaling pathways, such as the Cav-1/RhoA and Scr/Cav-1/EGFR/Akt signaling pathways, resulting in the prevention of fibronectin and collagen I production and alleviation of glomerulosclerosis (Peng et al., 2007; Zhang et al., 2007). In addition, MβCD can affect the signal transduction of ANG-II receptors and reduce glomerular mesangial hyperplasia (Adebiyi et al., 2014). Previous studies have shown that Cav-1 located on caveolae of glomerular ECs (HRGECs) mediates albumin entry into glomerular ECs and transcytosis, leading to proteinuria in glomerular diseases, whereas MβCD can reverse the abovementioned changes (Moriyama et al., 2015; Moriyama et al., 2017). In addition, Daidzein, another inhibitor of Cav-1 (Sharma et al., 2012; Gao et al., 2014), has been found to have anti-inflammatory, antioxidative stress, and renoprotective effects in the mice with AKI induced by cisplatin (Meng et al., 2017; Tomar et al., 2020). Furthermore, Daidzein can also reverse the pathological changes in diabetic rats and reduces urine protein, blood creatinine, and urea nitrogen by inhibiting the Cav-1/eNOS/NO pathway (Arya et al., 2011). In addition, Daidzein has a potential effect on diabetes, and its complications are mediate by regulating cellular metabolism, including glucose and lipid metabolism, as well as oxidative stress (Das et al., 2018). Daidzein improves hyperglycemia, insulin resistance, dyslipidemia, obesity, and inflammation (Das et al., 2018). These data suggest that Cav-1 inhibitors can affect the progression of kidney disease by regulating cellular metabolism, but the precise mechanism is still unclear.

#### 6.1.2 Traditional Chinese Medicine Extracts

Curcumin, as an extract from traditional herbal medicine, has multiple functions, such as antioxidation, anti-inflammatory, and antifibrosis activities (Santos-Parker et al., 2017; Tsuda, 2018; Kong et al., 2020). It can regulate the fibrosis process of kidney disease through Cav-1–related signaling pathways (Sun et al., 2017). Under high-glucose conditions, the Cav-1/TLR-4 signaling pathway mediates pro-inflammatory factors, such as TNF-α, IL-6, IL-1β, and MCP-1, to induce the reversal of podocyte damage in response to curcumin (Sun et al., 2014b). In addition, Curcumin can also inhibit the phosphorylation of Cav-1 under high-glucose conditions and alleviate the oxidative stress and apoptosis of podocytes, as well as the epithelial-mesenchymal transition (EMT) and proteinuria of podocytes, eventually delaying the progression of diabetic mice (Sun et al., 2014a; Sun et al., 2016).

Salidroside (SAL) is an active ingredient isolated from the traditional Chinese medicine rhodiola that has a protective effect on kidney diseases. Wu et al. found that SAL upregulates AMPK and downregulates Src kinase under high-glucose conditions, thereby inhibiting Cav-1 phosphorylation, inhibiting glomerular ECs (GECs) albumin endocytosis, and reducing albuminuria in diabetic mice (Wu et al., 2016). Xue et al. found that SAL can activate the Sirt1/PGC-1α axis to promote mitochondrial biogenesis and alleviate pathological changes in diabetic mice, as shown by decrease in urine albumin, blood urea nitrogen and serum creatinine (Xue et al., 2019). Furthermore, SAL inhibits the TLR4/NF-κB and MAPK pathways to prevent inflammation and fibrosis, protecting kidney function (Li et al., 2019). Previous studies have shown that Cav-1 regulates the TLR4 signaling pathway and participates in the inflammatory response of podocytes (Sun et al., 2014b). Whether SAL regulates the Cav-1/TLR4 pathway to reduce renal inflammatory damage requires follow-up research.

Catalpol was previously found to have neuroprotective effects in diabetic mice by increasing the expression of Cav-1 and PKC (Zhou et al., 2014). The review by Bai et al. summarized that the ability of catalpol to reduce kidney damage by improving lipid metabolism and IGF-1 signal transduction (Bai et al., 2019). On the basis of the function of Cav-1 in regulating lipid metabolism, catalpol may play a key role in kidney disease by regulating the expression of Cav-1.

#### 6.1.3 Rock Inhibitor: Fasudil

It has been reported that Cav-1 plays a key role in regulating the RhoA/Rock pathway, which is involved in inflammation and apoptosis in kidney disease (Peng et al., 2007; Wu et al., 2014; Nozaki et al., 2015; Zhao et al., 2015). Fasudil, a Rock inhibitor, blocks the VEGF/Src/Cav-1/signaling pathway to alleviate renal inflammation, glomerulosclerosis, and proteinuria in diabetic mice (Xie et al., 2013; Jin et al., 2015). In addition, Rock inhibitor also plays an important role in cellular metabolism. In high-fat–fed mice, fasudil can activate AMPK, thereby promoting lipid metabolism (Noda et al., 2014; Noda et al., 2015). Furthermore, in diabetic mice, the RhoA/ROCK/NF-κB signaling pathway is inhibited when pancreatic islets are transplanted into mice, and the production of podocyte inflammatory factors such as IL-6 and MCP-1, is reduced, reversing podocyte damage (Huang et al., 2021). Recently, the role of Rho family GTPases in regulating cell glucose metabolism and maintaining glucose homeostasis has also received increasing attention (Møller et al., 2019; Machin et al., 2021). Therefore, whether Rock inhibitors indirectly affect Cav-1–related signaling pathways to regulate kidney energy metabolism still needs to be addressed.

#### 6.1.4 Noncoding RNA

Many studies have shown that noncoding RNAs regulate the expression of Cav-1 and affect its downstream events. It has been found that miR-204 (Hall et al., 2014) and circAKT1 (Zhu et al., 2020) affect the progression of kidney disease by regulating Cav-1. Hall et al. found that, in VHL (−) RCC cells, miR-204 indirectly affects the transient receptor potential melastatin 3 (TRPM3)–induced autophagy by inhibiting the expression of Cav-1, which, in turn, affects the development of RCC (Hall et al., 2014). In addition, Zhu et al. found that circAKT1, which is highly expressed in clear cell RCC, promotes the proliferation and progression of renal cancer cells by upregulating the expression of Cav-1 by sponging miR-338-3p (Zhu et al., 2020). However, Mehta et al. recently found that *CAV1* deficiency in glomerular mesangial cells can inhibit miR299a-5p, which may posttranscriptionally regulate the expression of follostatin, thereby exerting an anti-renal fibrosis effect (Mehta et al., 2021). The above studies may indicate that noncoding RNAs are involved in the progression of Cav-1–mediated kidney disease.

## 7 Conclusion and Perspectives

**FIGURE 3:**
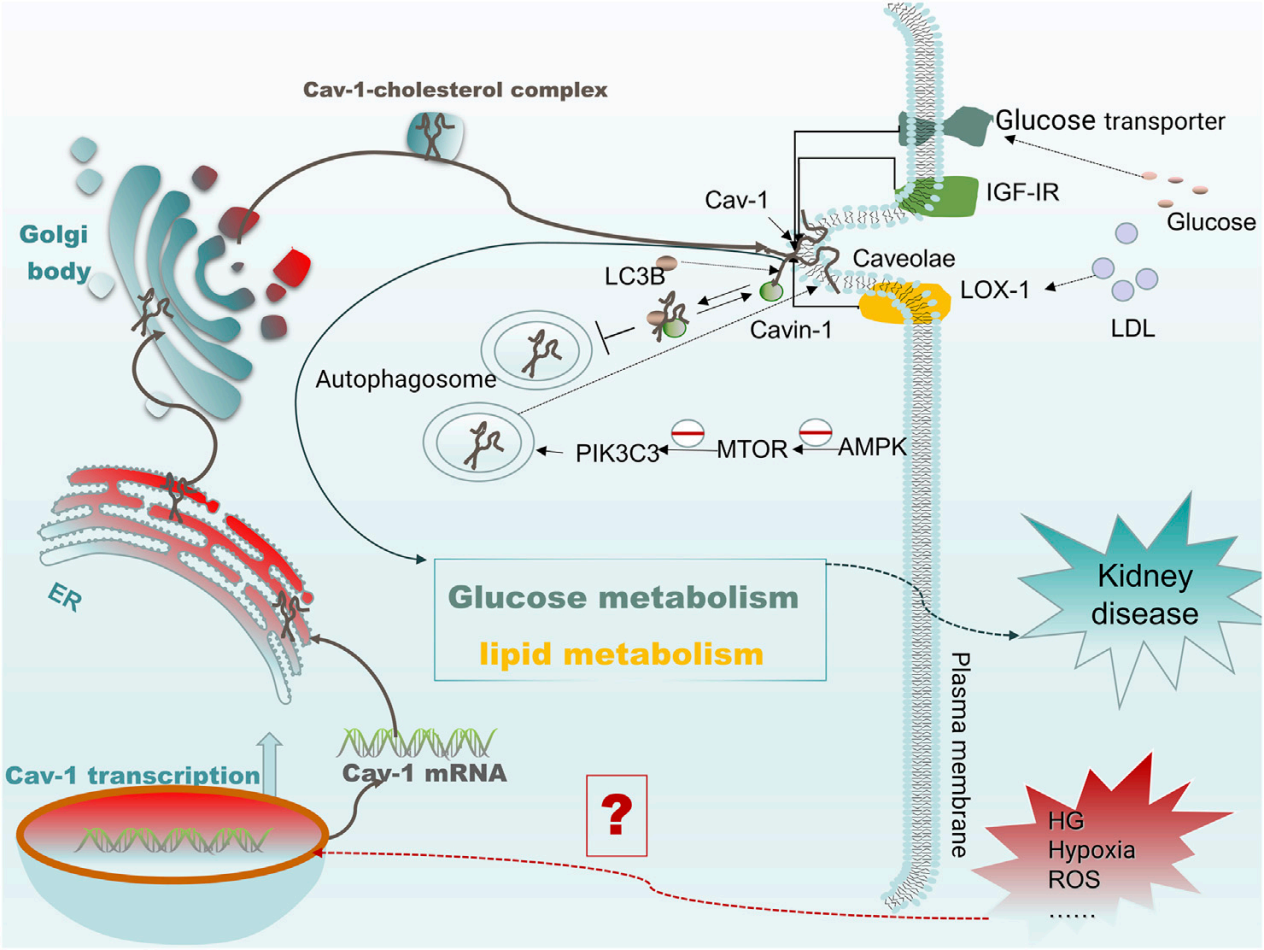
Cav-1 mediates protein membrane targeting and regulates cellular metabolism. When cells are subjected to harmful external stimuli (such as high glucose and oxidative stress), the transcription levels of *CAV1* increase, which promotes the formation of caveolae on the cell membrane. The expressed Cav-1 can then recruit receptors such as IGF-IR, glucose transporter, and LOX-1 to caveolae and bind there to Cav-1 and subsequently regulate glucose or lipid metabolism by mediating specific receptor signals. In addition, under high glucose conditions, blocking autophagy induced by the AMPK-MTOR-PIK3C3 pathway reduces the degradation of Cav-1 by autophagy, resulting in increased expression of cavin and Cav-1. The high expression of Cav-1 recruits LC3B and binds together to inhibit the formation of autophagosomes, further inhibiting autophagic degradation of Cav-1 and leading to an increase in the formation of caveolae, which eventually mediates increased endocytosis of low-density apolipoprotein.

Cav-1 is a metabolism-related membrane protein in a variety of cell types, which is involved in the pathophysiology of a variety of diseases by a large signaling network system. Despite recent studies showing robust evidence for the critical role of Cav-1 in metabolic disorders, oxidative stress, and autophagy, most of the studies indicated that Cav-1 is a potential therapeutic target in cancer and cardiovascular diseases, whereas few studies have been conducted in kidney disease. Because cellular metabolic homeostasis is critical in kidney disease, here, we propose that Cav-1 is closely linked to kidney disease through the regulation of cellular metabolism. When cells are subjected to stress and other stimuli, the expression of Cav-1 is increased, and it is then translocated to the cell membrane. At the membrane, it recruits specific receptors and molecules such as IGF-IR, glucose transporters, and LOX-1 into the caveolae, mediating downstream signal transduction and affecting cellular metabolism including glucose and lipid metabolism. Conversely, in vascular ECs treated with high glucose, autophagy induced by the AMPK-MTOR-PIK3C3 pathway is blocked to reduce the degradation of Cav-1 by autophagy, whereas the increased expression of Cav-1 inhibits the formation of downstream autophagosomes by recruiting and binding to LC3B, thus further inhibiting the autophagic degradation of Cav-1 and leading to an increase in caveolae formation. This process mediates the increase in endocytosis of low-density apolipoprotein and affects cellular metabolism (Bai et al., 2020). As mentioned above, Cav-1 may have a potential role in kidney disease by regulating cellular metabolism (Figure 3). In addition, we also describe Cav-1 and its related signaling molecules as potential therapeutic targets based on the use of related inhibitors and extract from traditional medicines in various kidney diseases. This review may open up new horizons for future investigations of the role of Cav-1 in kidney and other disease.
